# Canine transmissible venereal tumour established in immunodeficient mice reprograms the gene expression profiles associated with a favourable tumour microenvironment to enable cancer malignancy

**DOI:** 10.1186/s12917-021-03093-4

**Published:** 2022-01-03

**Authors:** Chiao-Hsu Ke, Hirotaka Tomiyasu, Yu-Ling Lin, Wei-Hsiang Huang, Hsiao-Hsuan Huang, Hsin-Chien Chiang, Chen-Si Lin

**Affiliations:** 1 Department of Veterinary Medicine, School of Veterinary Medicine, National Taiwan University, No. 1 Sec. 4 Roosevelt Rd., 10617 Taipei, Taiwan; 2 Department of Veterinary Internal Medicine, Graduate School of Agricultural and Life Sciences, The University of Tokyo, 1-1-1, Yayoi, Bunkyo-ku, Tokyo, 113-8657 Japan; 3 Agricultural Biotechnology Research Center, Academia Sinica, Taipei, 11529 Taiwan; 4 Graduate Institute of Molecular and Comparative Pathobiology, School of Veterinary Medicine, National Taiwan University, 10617 Taipei, Taiwan; 5 Industrial Development Graduate Program of College of Biological Science and Technology, National Yang Ming Chiao Tung University, Hsinchu City, 30068 Taiwan; 6 Laboratory 2612, Rekiin Biotech Inc., 114737 Taipei, Taiwan

**Keywords:** Tumour biomarkers, Infectious cancer, Canine tumour model, Immunoediting, cDNA microarray

## Abstract

**Background:**

Canine transmissible venereal tumours (CTVTs) can cross the major histocompatibility complex barrier to spread among dogs. In addition to the transmissibility within canids, CTVTs are also known as a suitable model for investigating the tumour–host immunity interaction because dogs live with humans and experience the same environmental risk factors for tumourigenesis. Moreover, outbred dogs are more appropriate than inbred mice models for simulating the diversity of human cancer development. This study built a new model of CTVTs, known as MCTVTs, to further probe the shaping effects of immune stress on tumour development. For xenotransplantation, CTVTs were first injected and developed in immunodeficient mice (NOD.CB17-Prkdc^scid^/NcrCrl), defined as XCTVTs. The XCTVTs harvested from NOD/SCID mice were then inoculated and grown in beagles and named mouse xenotransplantation of CTVTs (MCTVTs).

**Results:**

After the inoculation of CTVTs and MCTVTs into immune-competent beagle dogs separately, MCTVTs grew faster and metastasized more frequently than CTVTs did. Gene expression profiles in CTVTs and MCTVTs were analysed by cDNA microarray to reveal that MCTVTs expressed many tumour-promoting genes involved in chronic inflammation, chemotaxis, extracellular space modification, NF-kappa B pathways, and focal adhesion. Furthermore, several well-known tumour-associated biomarkers which could predict tumour progression were overexpressed in MCTVTs.

**Conclusions:**

This study demonstrated that defective host immunity can result in gene instability and enable transcriptome reprogramming within tumour cells. Fast tumour growth in beagle dogs and overexpression of tumour-associated biomarkers were found in a CTVT strain previously established in immunodeficient mice. In addition, dysregulated interaction of chronic inflammation, chemotaxis, and extracellular space modification were revealed to imply the possibly exacerbating mechanisms in the microenvironments of these tumours. In summary, this study offers a potential method to facilitate tumour progression and provide a niche for discovering tumour-associated biomarkers in cancer research.

**Supplementary Information:**

The online version contains supplementary material available at 10.1186/s12917-021-03093-4.

## Background

Cancer is the leading cause of death in both humans and dogs, but current tumour treatments usually achieve undesirable therapeutic efficiency, especially in some knotty cancers. Syngeneic murine models or immunodeficient mice have allowed the engraftment of human xenotransplantation in cancer research for decades [1]. However, rodent models have limitations and even lead to misinterpretations in evaluating the effectiveness of newly developed therapies, especially immunotherapies in human or veterinary medicine [2]. The anti-tumour efficacy of new immunotherapies cannot be evaluated in immunodeficient mice [1], indicating a need for an animal model with intact immunity. In addition to those in immunocompetent animals, canine cancers share many developmental and pathological similarities with their human counterparts. As companion animals, dogs live with humans and experience the same environmental risk factors for tumourigenesis. Moreover, outbred dogs are more suitable than inbred mice models for simulating the diversity of human cancer development [2, 3].

Canine transmissible venereal tumours (CTVTs) are contagious round cell neoplasms that are frequently located in the external genitalia of both genders and are transmitted by the transplantation of cells during coitus [4]. CTVTs can be allotransplanted using intact viable cells across the major histocompatibility complex (MHC) barriers within the same species and even in other members of the canine family, such as foxes, coyotes, and wolves [5, 6]. Since CTVTs are transplantable tumours, the application of CTVT transplantation in dogs as tumour models has been documented in immunology and tumour research [4, 7]. Thus, this study intended to determine the possible details or mechanisms between host immunity and CTVT, a tumour that can be grown in any immunocompetent dog. CTVTs usually spontaneously regress (the SR phase) after progressive growth (the P phase), during which the tumours secrete a high concentration of TGF-β, which suppresses the expression of MHC class I and II molecules in tumours [8]. These inhibitory immune activities describe the rapid tumour growth that occurs during the P phase. In contrast, during the SR phase, a high level of IL-6 is secreted by the tumour-infiltrating lymphocytes (TILs), antagonizes the effects of TGF-β, and restores the activities of IFN-γ [9]. Collectively, CTVTs spontaneously regress under an intact immune system with the IL-6 secretion from the TILs and the upregulation of the MHC class I and II molecules of tumours; the overexpression of MHC molecules triggers the immune system and eradicates the CTVTs.

Genomic instability is a fundamentally important feature of almost all cancers, and the tumour microenvironment (TME) can also contribute to significant genetic changes in tumours [10]. The genetic alterations in tumours are highly associated with the formation of biomarkers, which have become effective indicators of tumour categorization, malignancy, and prognosis [11]. Moreover, these markers can be used to detect the growth of tumours [12] and usually serve as the treatment targets of patients with cancers [13]. A previous study has demonstrated that CTVT cells being transplanted into a deficient immune environment, NOD/SCID mice, are permanently in the P phase (xenotransplanted CTVTs, XCTVTs, are unable to exhibit the SR phase) [14]. We have also successfully established the XCTVT model according to the previous study [15]. Furthermore, in current study, the XCTVT cells were harvested and inoculated into beagles, and the tumours developed in beagles are referred to as mouse-CTVTs (MCTVTs). Previous studies have showed that XCTVTs retain the morphology, histological features, and biological characteristics of CTVTs [14]. However, it remains unknown if XCTVTs change their growth or gene expression patterns when re-inoculated into beagles. Answering this question could provide important clues about how tumours adapt to different immune defense strengths. This study aimed to investigate the characteristics of persistent-P-phase CTVT cells (MCTVT) and naïve CTVT cells arising in healthy beagles and to probe whether surrendered TME (XCTVTs arising in the NOD/SCID mice) can reprogram the gene profiles and thereby expose potential tumour markers related to cancer malignancy.

## Results

### MCTVT becomes a more malignant tumour compared to CTVT

**Fig. 1:**
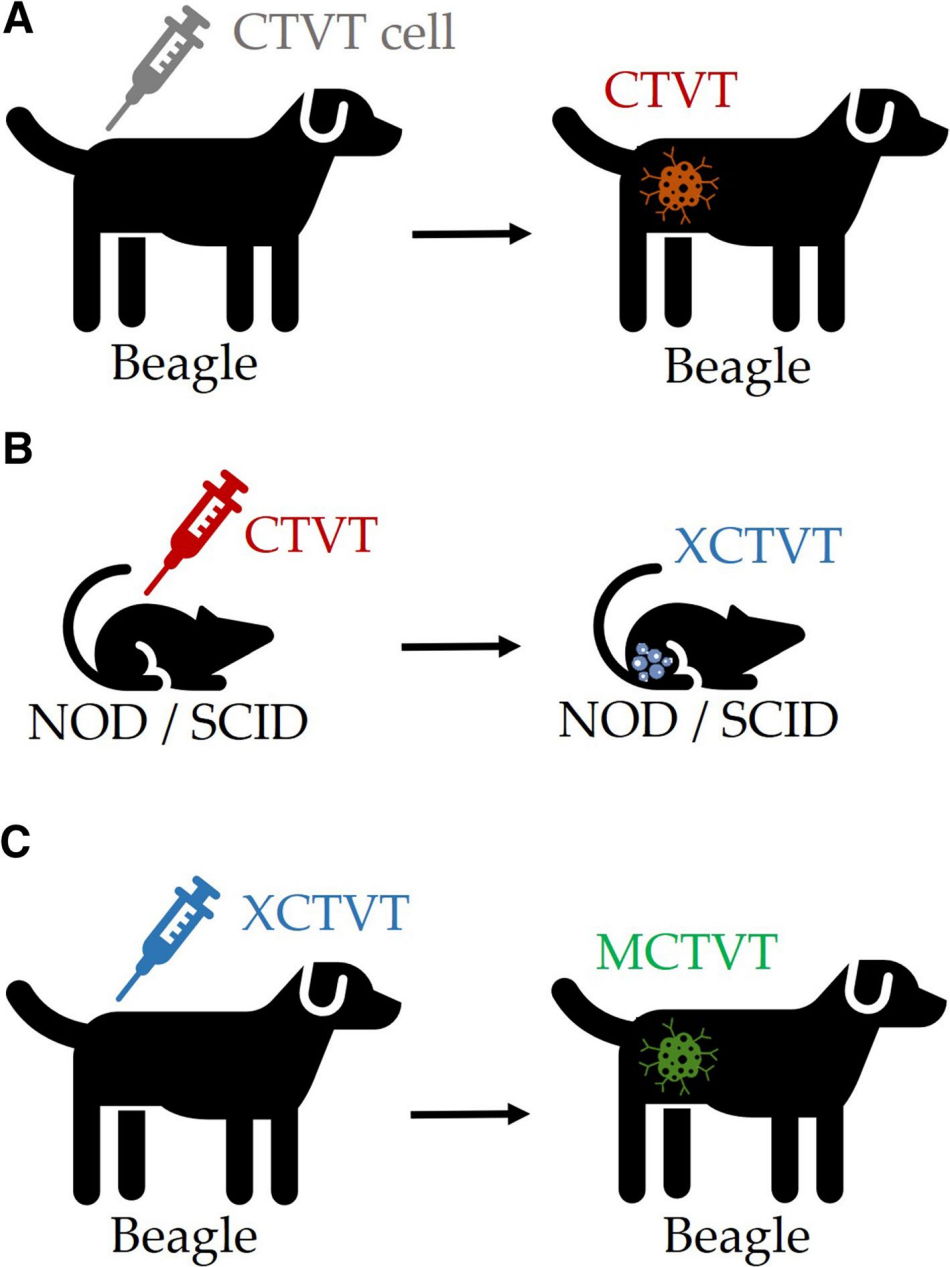
Schema of tumour model establishment. **A** To establish this canine animal model, primarily spontaneous canine transmissible venereal tumours (CTVTs) on the external genitalia of one male dog were used for the original transplantation. Dogs were inoculated subcutaneously with freshly prepared CTVT cells on their back; **B** CTVTs harvested from the tumour-bearing dogs were inoculated into immunodeficient NOD/SCID mice. Without immune cells attacking, the tumours grew in NOD/SCID mice and were called xenograft canine transmissible venereal tumours (XCTVTs); **C** Next, XCTVT cells were inoculated subcutaneously into beagles, and the tumours which grew on the beagles were defined as mouse canine transmissible venereal tumours (MCTVTs)

**Fig. 2:**
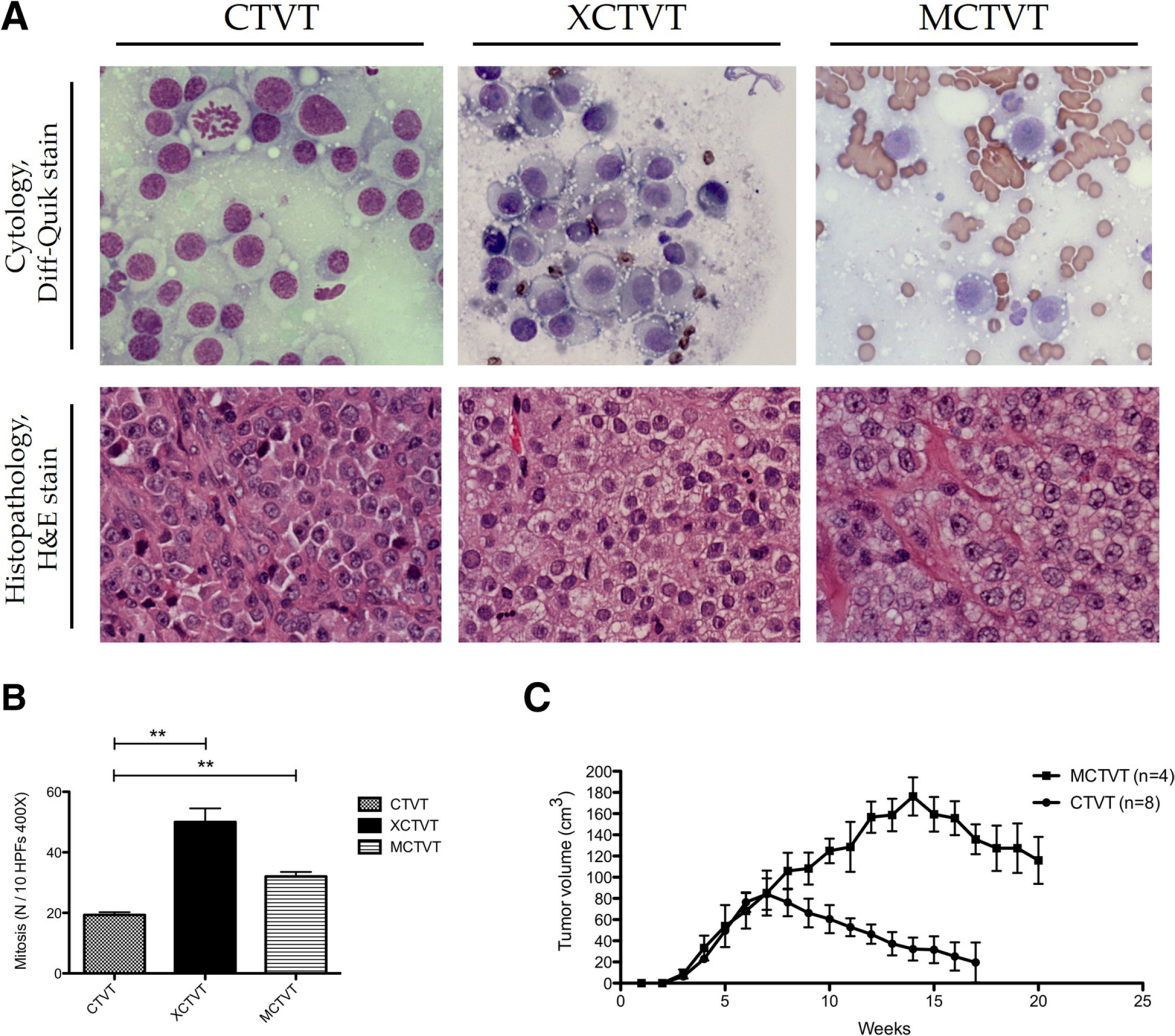
Cytology, histopathology, mitosis, and tumour growth curves of various tumours. **A** The three tumours shared similarities in cytological features, such as the moderate to low nuclei-cytoplasmic ratio, cytoplasmic vacuolization, and huge nucleoli in week 10; however, the intracytoplasmic vacuolization and size of the cells indicated greater malignancy of XCTVTs (top middle) and MCTVTs (top right) than of CTVTs (top left). Histologically, these three tumours were composed of round, oval or polyhedral cells with a moderate to small nuclei-cytoplasmic ratio. MCTVTs (bottom right) had a more progressive morphology, such as anisokaryosis and distinct borders of tumour cells than CTVTs (bottom left) and XCTVTs (bottom middle); **B** Mitotic counts of MCTVTs (32 ± 2.65) and XCTVTs (50 ± 7.81) were significantly higher than those of CTVTs (19.33 ± 1.53) (*n* = 3 in each group); **C** Tumour growth curves showed that MCTVTs were more aggressive than CTVTs in dogs. All data are presented as mean ± SD and statistical significance was calculated by unpaired *t* test (**, *p* < 0.01)

CTVTs in the external genitalia of one male dog were used for the original transplantation. Dogs were inoculated subcutaneously with freshly prepared 10^8^ CTVT cells in their backs for XCTVT preparation (Fig. 1A). After 10 weeks, XCTVTs that developed progressively in canids were transplanted in immunodeficient mice (NOD/SCID). The XCTVTs were palpable after injection and achieved diameters of approximately 2 cm in about 4 weeks (Fig. 1B). Then the tumour masses were harvested and subcutaneously inoculated into beagles. These tumours arising in the beagles are referred to herein as MCTVTs (Fig. 1C). The cytological features of the XCTVTs (Fig. 2A, top middle) and MCTVTs (Fig. 2A, top right) were similar to those of typical CTVTs (Fig. 2A, top left), but the intracytoplasmic vacuolization in XCTVTs and MCTVTs was more prominent than that of CTVTs, indicating the more malignant features of MCTVTs and XCTVTs. Histologically, these three tumours were composed of round, oval, or polyhedral cells divided into small islets by fine connective tissues in H&E stain. The nuclei-cytoplasmic ratio was moderate to low. The nuclei exhibited coarse clumping of chromatin and contained a huge nucleolus, which is one of the known histopathological characteristics of this tumour. However, MCTVTs (Fig. 2A, bottom right) had more progressive morphology, such as anisokaryosis and distinct borders of tumour cells, than those of CTVTs (Fig. 2A, bottom left) and XCTVTs (Fig. 2A, bottom middle). In addition, the mitotic counts were higher in MCTVTs (32 ± 2.65 per 10 consecutive high-power fields) and XCTVTs (50 ± 7.81 per 10 consecutive high-power fields) than in CTVTs (19.33 ± 1.53 per 10 consecutive high-power fields) (Fig. 2B). The results of mitosis reflected the pattern of the tumour growth curve. After approximately 7 weeks of development, the CTVTs spontaneous regressed, as expected; however, the MCTVTs showed uncontrolled progressive growth until the 14th week. These results indicated that MCTVTs were significantly more aggressive than CTVTs (Fig. 2C). In summary, MCTVTs, which originated from a deficient immune environment, were found to be highly aggressive tumours of greater malignancy than CTVTs. The divergent phenotypes of CTVTs and MCTVTs raise the question of how MCTVTs gain the ability to survive aggressively.

### Transcriptome expression profiles are reprogrammed in MCTVTs

**Fig. 3:**
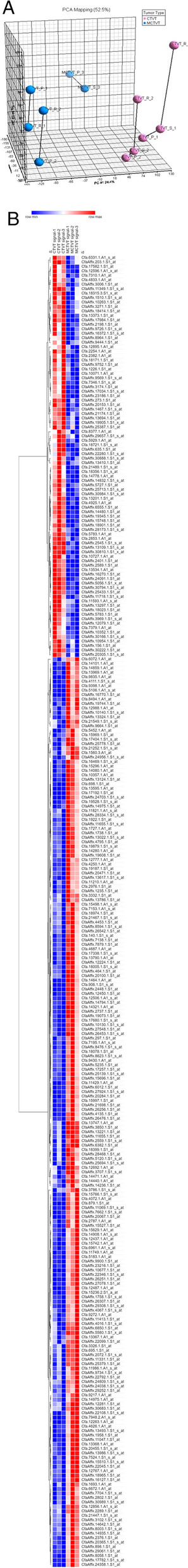
Gene expression profiles of CTVTs and MCTVTs. Interaction of differentially expressed genes (DEGs). **A** Gene expression profiles of CTVTs and MCTVTs developed using PCA mapping. In PCA mapping, distinct distributions of the expression profiles of MCTVTs and CTVTs were identified. Blue dots represent MCTVT gene expression profile and pink dots imply distribution of CTVT gene expression; **B** Heatmap of representative gene expression between CTVTs and MCTVTs by hierarchical clustering

To probe the potential mechanisms of malignant MCTVTs, the transcriptome expression profiles of CTVTs and MCTVTs were analyzed by Affymetrix Canine Genome 2.0 expression array. The results revealed that CTVT and MCTVT samples were distributed into two distinct groups in principal components analysis (PCA) mapping (Fig. 3A), indicating meaningful differences in gene expressions between the two tumours. We then proceeded to investigate malignant signature by comparing the gene expression profiles of these two tumours. The differentially expressed genes (DEGs) were filtered by the following criteria. First, the genes must be presented in all the examined chips (triplicate gene expression profiles in CTVT and MCTVT, respectively). Second, the DEGs were selected above the 2-fold or below the 0.5-fold difference between the gene expressions of CTVTs and MCTVs in the P phase with a *p*-value < 0.05 [16]. Total 173 DEGs were found under the criteria. The expression levels of 136 genes were significantly higher (above the 2-fold change) in MCTVTs than in CTVTs (Additional file 1), and those of 37 genes were significantly lower (below the 0.5-fold change) in MCTVTs (Additional file 2). DEG heatmaps were generated separately for the two tumours to show that the gene expressions of MCTVTs and CTVTs were distinct (Fig. 3B). These results indicated that MCTVTs had shifted their tumour transcriptome schema from the CTVT origin after passing through the microenvironment in immunodeficient mice, and the DEGs possibly participated in the tumourigenesis of MCTVT.

### DEGs in MCTVTs disclosed the tumour malignancy related to chemotaxis, inflammation, and focal adhesion in the tumour microenvironment

**Fig. 4:**
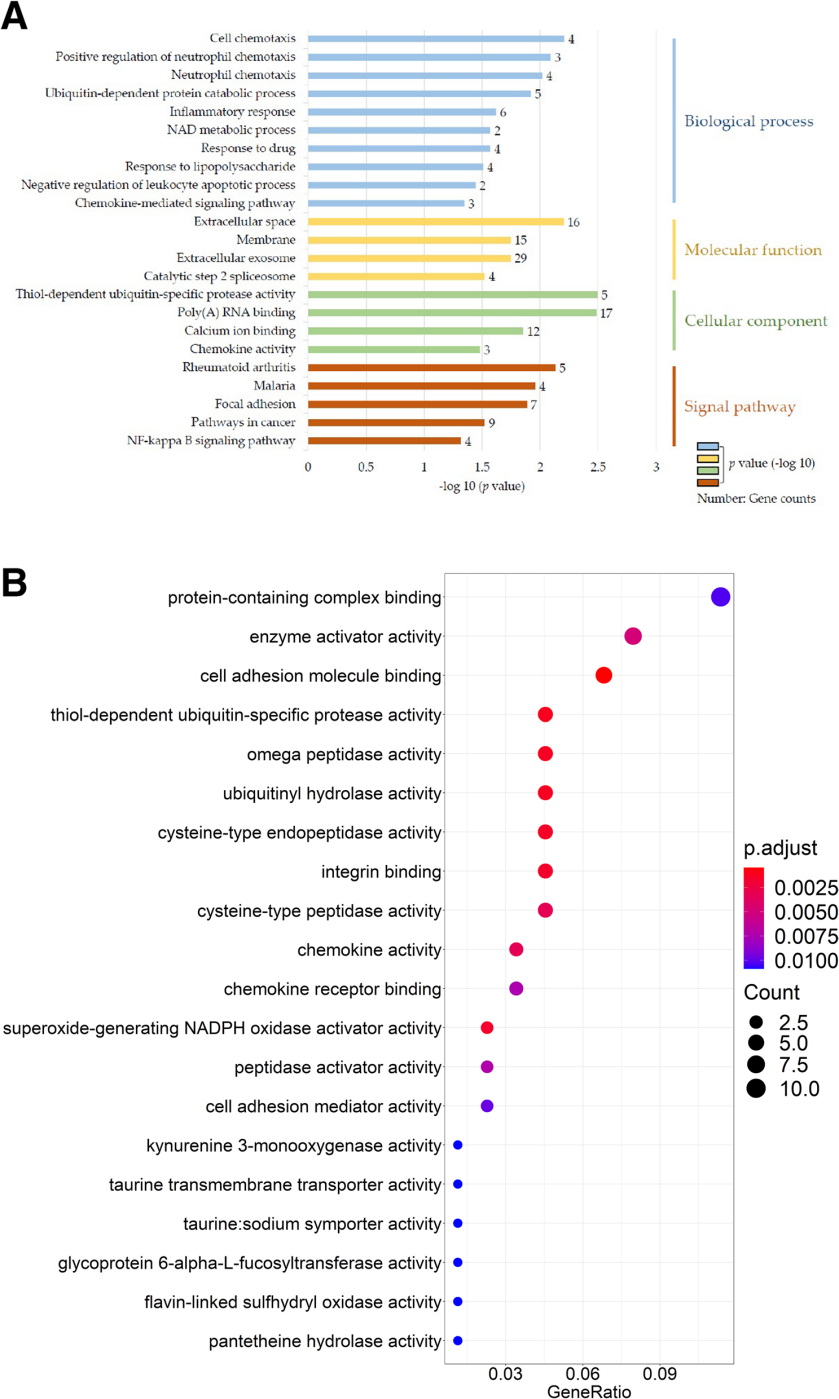
Enriched Gene ontology (GO) term, Kyoto Encyclopedia of Genes and Genomes (KEGG) pathways, and Gene Set Enrichment Analysis (GSEA) analysis of the differentially expressed genes (DEGs). **A** Representative enriched GO terms, including biological process (BP), cellular component (CC), molecular function (MF), and KEGG pathway enrichment analysis, were carried out for the DEGs. The green bar represents -log 10 (*p* value) and the numbers beside the bars present the gene numbers involved in the certain signal pathways. **B** Top 20 BP of GSEA analysis. The plots represent the gene counts and the colours of the plots were the *p* value of the identified BP

**Fig. 5:**
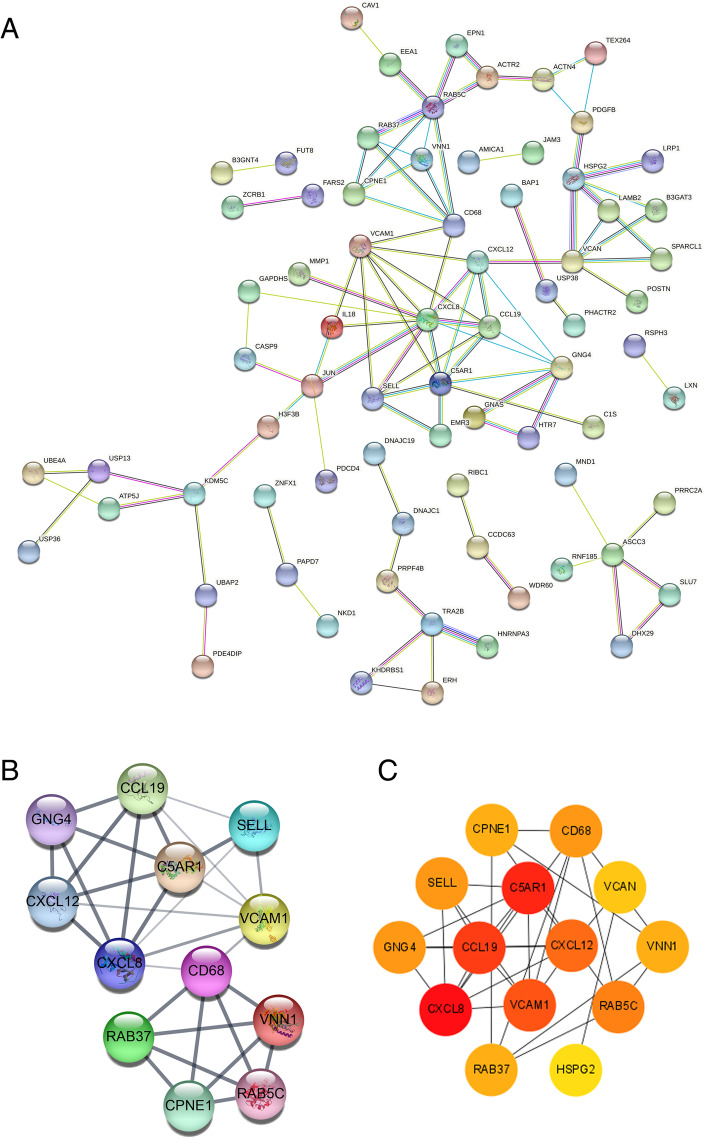
Protein-protein interaction (PPI) network and analysis of the hub genes of differentially expressed genes (DEGs). **A** PPI analysis of using STRING networks reveals the tight interaction between the DEGs. A thicker edge indicates a higher confidence score of the interaction; **B** Clustering analysis of the PPI network revealed the most important module, identified using Cytoscape (MCODE plugin); **C** The hub genes were identified by the cytoHubba tool in Cytoscape. The selected nodes are shown with a color scheme from essential (red) to crucial (yellow)

**Fig. 6:**
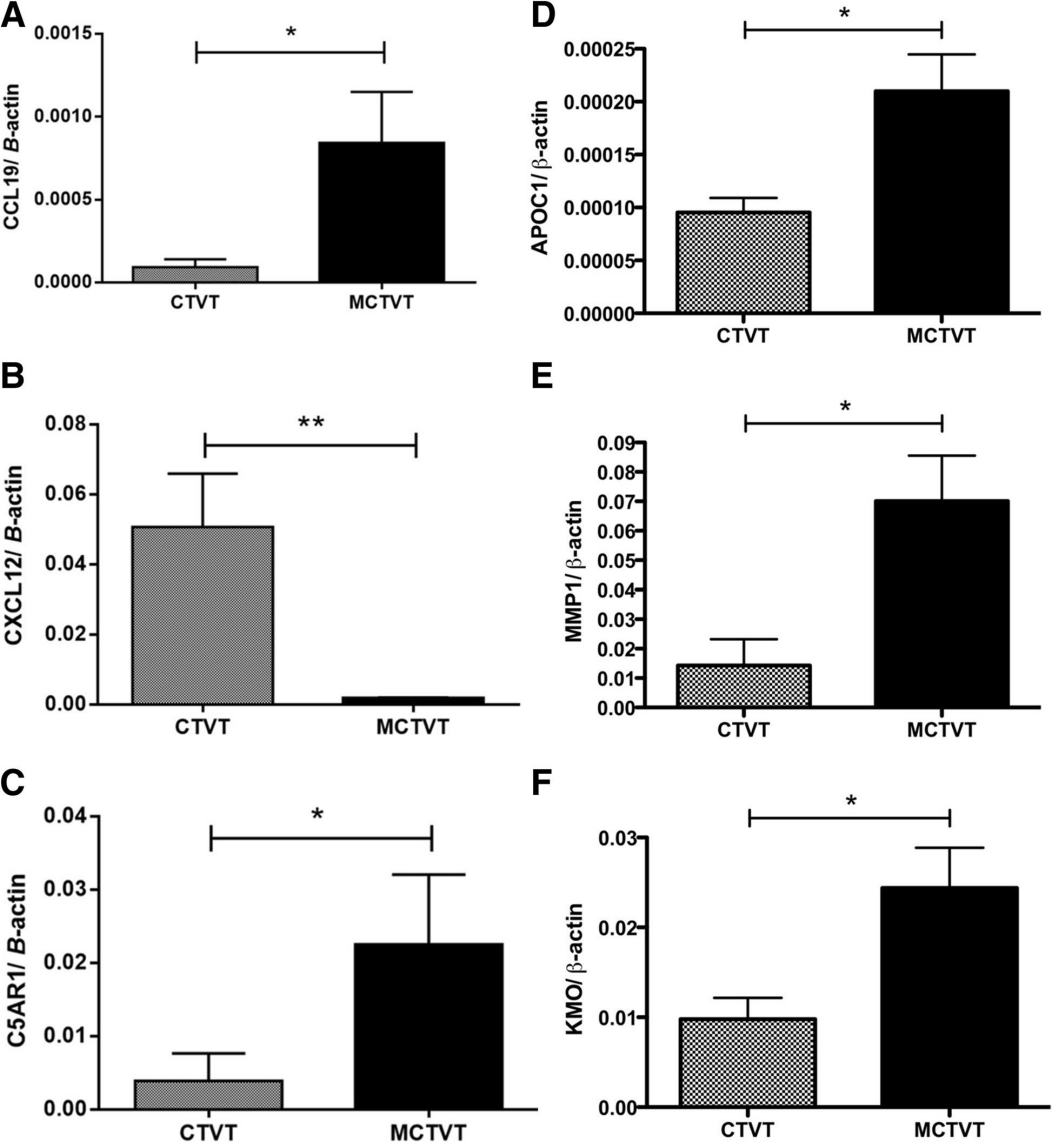
Hub genes correlated to chemotaxis and representative malignant biomarker genes were validated by quantitative PCR. The gene expression levels of (**A**) *CCL19*, (**B**) *CXCL12*, and (**C**) *C5AR1* between CTVT and MCTVT were consistent with cDNA microarray data. Gene levels of (**D**) *APOC1*, a tumour promoter, (**E**) *MMP1*, which was overexpressed in various cancers, and (**F**) *KMO*, which promoted tumour progression, were significantly increased in MCTVTs compared with CTVTs. All data are presented as mean ± SD and statistical significance was calculated by unpaired *t* test (*, *p* < 0.05)

To depict the possible mechanisms contributing to the malignancy of MCTVT, the DEGs in CTVTs and MCTVTs were next imported into the functional enrichment analysis of Gene Ontology (GO) using the Database for Annotation, Visualization, and Integrated Discovery (DAVID) and the Kyoto Encyclopedia of Genes and Genomes (KEGG) for analysis. GO analysis of the DEGs identified 10 biological process (BP), 4 cellular component (CC), and 4 molecular function (MF) GO terms as significantly enriched (*p* < 0.05). KEGG pathway analysis revealed 6 significantly enriched KEGG pathways (*p* < 0.05). Most of the GO term and KEGG pathway analyses were related to “cell chemotaxis”, “chemokine activity”, “inflammatory response”, “NF-kappa B pathways”, “focal adhesion”, “pathways in cancer”, and other cancer-related functional pathways (Fig. 4A). To further verify our results, we now further use Gene Set Enrichment Analysis (GSEA) software (https://www.gsea-msigdb.org/gsea/index.jsp) [17, 18], the DEGs analytic method improving the possible bias generated by a fold-change selection. The GSEA output results also revealed that DEGs were highly correlated to cell adhesion, integrin binding, chemokine activities, and other tumour-related signal pathways (Fig. 4B). To clarify the interactions of the DEGs, the Search Tool for the Retrieval of Interacting Genes/Proteins (STRING) networks were employed to analyze their correlations (Fig. 5A). The most crucial module, which was scored at 5.455 and included 12 nodes, was obtained using Cytoscape (MCODE plugin) (Fig. 5B). To select the hub genes among all the DEGs, the CytoHubba plugin was employed and selected 14 hub genes (Fig. 5C); moreover, 12 of the 14 were consistent with their enrichment in the top module analyzed by MCODE. Four of the 14 hub genes, namely, *C5AR1*, *CXCL12*, *CCL19*, and *VCAM1*, participated in numerous enriched functions, such as cell chemotaxis, regulation of the function of neutrophils, and NF-kappa B pathways, which potentially facilitated the growth of MCTVTs and establishment of the tumour-friendly microenvironment. The gene expressions of *C5AR1*, *CXCL12*, and *CCL19* (Fig. 6A – C) were further validated using quantitative PCR. The results were consistent with the microarray data, which indicates that with the different expressions of these hub regulators, MCTVT displaying a more malignant counterpart could result from the interaction between host immunity and the tumours. One of the most important reasons should be the significantly different gene expression profiles between CTVT and MCTVT. The altered genes and the enriched signalling pathways make MCTVT more malignant and the new growth environment also had impacts on the MCTVT development. These findings possibly explain the mechanisms of MCTVT malignancy and echo with the bioinformatics data, which suggested that DEGs in MCTVTs disclosed the tumourigenesis related to chemotaxis, inflammation, and focal adhesion.

### Tumour-related biomarkers are overexpressed in MCTVTs

MCTVTs developed progressively and exhibited a significantly greater malignant histopathological feature than CTVTs did. Three well-known malignant genes or therapeutic biomarkers, *APOC1* (Fig. 6D), *MMP1* (Fig. 6E), and *KMO* (Fig. 6F), were overexpressed in MCTVTs and ranked in the top 20 upregulated DEGs from this study (Additional file 1). We validated the gene expression of these 3 genes and other randomly selected genes (Additional file 3) by quantitative PCR, and the results were consistent with the microarray data. It is reported that high expression of tumour biomarkers, namely, *APOC1*, *MMP-1*, and *KMO*, leads to poor prognosis in cancer patients or tumourigenesis, which is in line with our findings in MCTVTs and highlights the importance of the overexpression of tumour-related biomarkers resulting from the surrendered immune environment.

## Discussion

CTVTs are naturally occurring tumours that are transmitted during copulation as intact tumour cells and affect the external or internal genitalia in canids. CTVT is the oldest known somatic cell lineage, as it first affected a dog that lived about 11,000 years ago [19]. As it has been widely used as a tumour model in numerous investigations of immunology and TME, including cytokine and tumour-infiltrating lymphocyte studies, CTVT is an ideal animal model for research into preclinical cancer immunotherapies [5, 19, 20]. The genome of CTVTs has recently been sequenced, revealing that CTVT is remarkably stable and has less genetic diversity; these unique characteristics allow the tumours to become long-living in nature [19, 21]. In addition, the long interspersed element (LINE) insertion is found specifically and constantly near the c*-Myc* gene of the CTVT cell and has been applied as a diagnostic marker for CTVT [22]. Since MCTVTs displayed a more malignant growth pattern and higher mitotic counts, MCTVTs and CTVTs share relatively high similarity in cytological and histopathological features (Fig. 2). For instance, MCTVTs and CVTVs consist of solitary or multiple tumour cell nodules divided by fine connective tissues into small islets. The two tumours are round to ovoid with characteristic large nucleoli and sometimes show prominent vacuolated cytoplasm and huge nucleoli in cytology. Also, the LINE gene fragment near the *c-Myc* gene characteristics are also confirmed in CTVTs and MCTVTs (Data not shown). Though the CTVT genome is highly conserved in canids [23], we first revealed that once CTVT xenotransplantation was performed in immunodeficient mice, the tumour exhibited aggressive progression (Fig. 2) and altered transcriptome profiles (Fig. 3).

The 3 identified hub genes (Fig. 5C) in MCTVT, namely, *CCL19*, *CXCL12*, and *C5AR1*, participated in the top 1 and 2 (ranked by *p* value) enriched biological processes, “cell chemotaxis (Fig. 4, *p* = 0.0062)” and “positive regulation of neutrophil chemotaxis (Fig. 4, *p* = 0.0081)”, as well as in several cancer-related KEGG pathways. Chemotaxis played a vital role in tumour formation and remodeling of the TME [24], where aggressive tumours can turn their surroundings into a comfortable environment that better meets their growth needs [25]. Tumour growth, immune system, tumour invasion, and metastatic progression are regulated by this complicated chemokine network [26, 27]. In the current study, *CXCL12* maintained a 0.29 fold-change in MCTVTs, while loss of *CXCL12* promotes tumour migration to the liver, bone marrow, and lung [28]. Expression of *CCL19* (which expressed a 2.6-fold-change in MCTVT) and *C5AR1* (which expressed a 2.28-fold-change in MCTVT) is highly associated with the advanced tumour stage and promotes cell proliferation in several cancers, leading to poor patient outcomes [29, 30]. Together, the synergic effects of the hub genes, *CCL19*, *CXCL12*, and *C5AR1*, promote chemotaxis, tumour proliferation, and even metastasis, which are in line with previous studies and evidenced by the progressive MCTVT growth (Fig. 2C). These results indicated that the malignant TME of MCTVTs might lead to transcriptomic alterations followed by the disclosure of potential cancer biomarkers [11] as well as therapeutic targets [13]. The identified enriched-KEGG pathways were highly correlated to the tumour-promotion, such as focal adhesion and NF-kappa B. These vital pathways were highly correlated to cell growth. For example, the cells started to migrate or grow by the activation of focal adhesion [31]; furthermore, NF-kappa B triggers the tumour cells migration and metastasis [32]. Cell adhesion plays a vital role in cancer development, which the alterations in the adhesion properties of tumours were highly correlated to the recurrent and progression of cancers [33]. Several studies have also shown that integrin binding-related biological processes resulted in cancer metastasis, drug resistance, and cancer stem-like properties [34, 35]. Chemotaxis played a vital role in tumour formation and remodelling of the TME [24], where aggressive tumours can turn their surroundings into a comfortable environment that better meets their growth needs [25]. Tumour growth, immune system, tumour invasion, and metastatic progression are regulated by this complicated chemokine network [26, 27]. In summary, the DEGs participated in significantly biological functions, which promoted the development of MCTVT and facilitated the malignancy of the TME.

After surviving in the immunocompromised hosts, MCTVTs expressed numerous tumour-associated biomarkers (Fig. 6), including 3 well-known malignant genes or therapeutic target candidates, *APOC1*, *MMP-1*, and *KMO*. *APOC1* is overexpressed in colorectal cancer (CRC) patients, and a high level of *APOC1* indicates a poor prognosis. In addition, *APOC1* has been proven as a biomarker in CRC [36]. Overexpression of *MMP-1* contributes to invasion and metastasis in breast cancer, and it potentially serves as a diagnostic marker and target for breast cancer [37]. *KMO* is a novel prognostic marker or oncogenic protein in human hepatocellular carcinoma [38] and triple-negative breast cancer [39, 40], suggesting that it is a promotor for tumour progression. Our previous studies also proved that *KMO* is overexpressed in canine mammary tumours (CMT) and canine melanoma and plays an oncogenic role in tumour development [41, 42]. These results showed that the deficient immunity could modify the transcriptome as well as give the growth advantage of the tumour cells.

Tumour biomarkers have become effective indicators of tumour categorization, malignancy, and prognosis [11]. In addition to the detection of tumour growth [12], tumour biomarkers often serve as the treatment targets in the development of immunotherapies [13]. This study used a CTVT model to show that, when tumours are implanted in immunodeficient mice, the relief of host immune stress will enable tumour cells to reprogram their transcriptomes and re-sculpt the tumour microenvironment. Simultaneously, through this model of tumour–host immunity interaction, the current study provides several potential tumour biomarkers for cancer monitoring, to further dissect their tumorigenic roles may provide the niche for the development of cancer target therapy.

## Conclusion

CTVT is considered as one of the ideal animal models for cancer research, especially for investigating the interaction between host immunity and cancer progression. This study first demonstrates that host immune–tumour microenvironment interplay can remodel the transcriptomic profiles of CTVTs, thereby inducing the altered expressions of tumour-related biomarkers, which should serve as objective indicators for the prediction of tumour development or grading, and even be designed as therapeutic candidates for cancer immunotherapies. Though further evidence is needed, our data suggested that tumour-related biomarkers which were hidden by the cancers could be uncovered by just inoculating the cancer cells into immunocompromised mice. Collectively, the results provide a potential solution to discover tumour-related biomarkers for cancer research.

## Methods

### Establishment of animal models

Healthy 1- to 2-year-old beagles were purchased from Lasco Co., Ltd. (Taiwan) in this research. Dogs were fed with commercially available canine food (Star Pro, TX, USA) and offered water by automatic water suppliers ad libitum. All dogs were dewormed regularly and vaccinated against canine distemper, leptospirosis, parvovirus, and canine hepatitis. A spontaneous CTVT on the external genitalia of a male dog was used for the original transplantation. Tumours were processed following a protocol proposed by Hsiao et al. with modifications [5]. Briefly, after tumour masses were crushed with a stainless-steel mesh and filtered once through two pieces of gauze (pore size: 190 μm), a tumour cell suspension was developed. The tumour cells were suspended in ATCC-formulated RPMI-1640 medium supplemented with 10% FBS (Invitrogen), streptomycin (100 ng/ml, Invitrogen), and penicillin (100 U/ml, Invitrogen). To isolate CTVT cells, the proportion of cell suspension to 42% Percoll™ (GE Healthcare Bio-Sciences Corp., USA) was 1:2. After centrifugation at 820 g for 30 min at 4 °C, purified CTVT cells deposited at the interface between the suspension medium and Percoll™ were harvested and washed three times with suspension medium. Dogs were inoculated with 1 × 10^8^ freshly well-prepared tumour cells subcutaneously in their backs. The tumour volumes were measured with calipers twice per week and calculated as π × length × width × thickness / 4 (cm^3^). Tumours were classified into different stages based on the tumour growth curve. The P phase was defined as the tumour volume consistently increasing. As tumours achieved stability, they were regarded as the S phase. Last, the R phase was defined as tumours gradually shrinking in volume.

6- to 8-week-old NOD/SCID mice were obtained from the Laboratory Animal Center, College of Medicine, National Taiwan University, Taipei, Taiwan. All mice were kept in specific pathogen-free barrier facilities at the Department of Veterinary Medicine, School of Veterinary Medicine, National Taiwan University, Taipei, Taiwan, and subcutaneously inoculated with 1 × 10^8^ viable CTVT cells on bilateral flanks. After sacrifice, the XCTVT tumours were harvested from these NOD/SCID mice and inoculated into others. In this study, mice were sacrificed due to ulcerations at the tumour site or tumour diameters exceeding 20 mm. After two serial passages in NOD/SCID mice, 1 × 10^8^ viable XCTVT cells were grafted into beagles subcutaneously and then serially passaged in dogs as MCTVT. The transplantation methods were the same as those for CTVT described above. The MCTVT masses and cells were collected for further investigation. All beagles were maintained at the Department of Veterinary Medicine, School of Veterinary Medicine, National Taiwan University, Taipei, Taiwan, following the guidelines of the Institutional Animal Care and Use Committee (IACUC No. NTU98-EL-00002). Research was carried out in compliance with the ARRIVE guidelines.

### Tumour sample collection and preparation

CTVT and MCTVT samples were surgically excised from tumour-bearing beagles, and XCTVT-bearing mice were euthanized with isoflurane and cervical dislocation to obtain their tumours. Single-cell suspensions were prepared from the tumour samples following the procedures described above. Tumour cell smears were stained with Diff-Quik stain (Dade Behring, USA) for subsequent cytological examination [43]. For histopathological analysis, tumours were fixed with 10% buffered formalin for 24 h and embedded in paraffin. Deparaffinized tissue sections (4 μm thick) were stained with hematoxylin and eosin and the mitotic index was determined by enumerating mitotic figures in 10 consecutive fields at 40x objective magnification in each sample. Slides were independently and separately scored by two board-certified veterinary pathologists from the NTU veterinary hospital who were blinded to the experiment.

### RNA extraction

Total RNA was extracted using TRIzol reagent (Invitrogen Life Technologies, USA) according to the manufacturer’s instructions. Briefly, tumour tissues were ground by mortar and pestle, resuspended in TRIzol reagent by vortex, and incubated at room temperature for 10 min. Following chloroform extraction, RNA was precipitated with isopropanol. The precipitated pellet was washed with 70% ethanol, dried in a vacuum chamber, and resuspended in diethylpyrocarbonate-treated water (DEPC-water). For Affymetrix analysis, total RNA samples were purified using RNeasy mini kits (Qiagen, Valencia, CA, USA) following the manufacturer’s protocol. Briefly, total RNA was resuspended in 350 μl buffer RLT and 250 μl 99% ethanol was added to the homogenized lysate by pipetting. Six hundred microliter RNA suspension was placed into an RNeasy mini spin column installed with an RNeasy membrane sitting in a 2-ml collection tube and centrifuged at 10,000 rpm for 15 s. The column was washed with RPE with centrifugation. RNA adhered to the RNeasy membrane was eluted with 40 μl of RNase-free water at 10,000 rpm for 1 min and then analysed on 1% denaturing agarose gel.

### Oligonucleotide microarray analysis

cDNA microarray was performed using GeneChip® Canine Genome 2.0 Array (Affymetrix, Santa Clara, CA, USA). For sample preparation, RNA was reverse-transcribed in the presence of a T7-(dT) 24 primer using a One-cycle cDNA Synthesis kit (Affymetrix). The cDNA product was then purified and transcribed in vitro with biotin-labelled ribonucleotides (IVT Labelling Kit; Affymetrix). The biotinylated DNA was then fragmented to 200 nucleotides or less, heated at 99 °C for 5 min, and hybridized for 16 h at 45 °C for the GeneChip® Canine Genome 2.0 Array, which contained 42,860 *Canis familiaris* probe sets for > 20,000 predicted genes. The GeneChip® was subsequently washed and developed according to the amplification staining protocol provided by Affymetrix and then scanned using an Affymetrix GeneChip® Scanner 3000. Affymetrix GeneChip® Operating Software, version 1.4, was used to quantify the expression levels of the genes represented on the GeneChip® Canine Genome 2.0 Array. Probe pairs were scored as positive or negative for the detection of a specific gene sequence by comparing signals from the perfect match and mismatched probe features. The number of probe pairs meeting the default discrimination threshold (= 0.015) was used to assign a call of absent (A), present (P), or marginal (M) for each assayed gene, and a *P* value was calculated. PCA reduced the dimensionality of the data (which included the expression profile of 19,000 genes) to three dimensions in order to visualize the similarity of the log-transformed expression ratios of the genes. For heatmap and hierarchical clustering, representative DEGs were chosen in gene expression profiles from triplicate CTVT and MCTVT samples.

### Quantitative PCR analysis of mRNAs

RNA was digested with DNase I (Fermentas, Canada) to remove genomic DNA. DNaseI was dissolved in 10x reaction buffer (Fermentas, Canada) with MgCl_2_ (Fermentas, Canada), 1 U/μL of DNaseI was added per 1 μg of RNA, and the mixture was incubated for 30 min at 37 °C. DNaseI activity was arrested by the addition of 1 μL of 25 mm EDTA (Fermentas, Canada), followed by incubation at 65 °C for 10 min. The RNA was denatured at 70 °C for 10 min and chilled on ice for 10 min, following which 4 μl of 5x first strand buffer (Invitrogen, Carlsbad, USA), 1 μl of 10 mM dNTPs, 2 μl of 100 mM DTT (Invitrogen, USA), 1 μl of RNase-free water, and 2 μl of SuperScript II RT (Invitrogen, USA) were added to the RNA solution. The RT reaction was carried out at 42 °C for 2 h in a Mastercycler Personal (Eppendorf, Germany). Quantitative PCR was performed (in triplicate) using SYBR Green PCR Master Mix (Bio-Rad, California, USA) according to the manufacturer’s instructions and a qPCR machine (Bio-Rad, California, USA). The primer sequences were designed using Primer 3 and are listed in additional file 4. The relative mRNA amount in each sample was calculated based on its threshold cycle in comparison with the threshold cycle of the housekeeping gene, β-actin. The results were presented as 2^-(Ct of target gene – Ct of housekeeping gene)^, (2^-DCT^), in arbitrary units and analysed using IQ5 analysis software (Bio-Rad, USA).

### Bioinformatics analysis

Identified DEGs were imported into DAVID (http://david.abcc.ncifcrf.gov/) to investigate the involvement of these genes in various BP, CC, and MF [44]. DAVID was used to carry out KEGG pathway analysis to reveal correlated pathways of DEGs [45]. Gene Set Enrichment Analysis (GSEA) software (https://www.gsea-msigdb.org/gsea/index.jsp) [17, 18] was employed to generate the BP. Morpheus (https://software.broadinstitute.org/morpheus/) was illustrated to generate clustered heat map for comparison of the representative DEGs [46], and the STRING networks (http://string-db.org) was employed to evaluate the interactions among the identified DEGs [47]. The results of STRING analysis were further analyzed by Cytoscape v.3.8.2 [48]. Molecular Complex Detection (MCODE), a plugin in Cytoscape, was used to identify the important modules in the PPI network. The default parameters were “degree cutoff = 2”, “node score cutoff=0.2”, “k-score = 2”, and “maximum depth = 100” [49]. To obtain the core genes, hub genes were further selected using the cytoHubba plugin [50].

### Statistical analysis

All data are presented as mean ± SD. Student’s *t*-test (two-tailed) was used to determine the statistical significance of differences and a *p* value < 0.05 was regarded as a significant difference (*, *p* < 0.05; **, *p* < 0.01; ***, *p* < 0.001; ****, *p* < 0.0001).

## Supplementary Information

**Additional file 1.** The 136 up-regulated genes (≥ 2-fold) in MCTVTs in comparison with CTVTs.

**Additional file 2.** The 37 down-regulated genes (≦ 0.5-fold) in MCTVTs in comparison with CTVTs.

**Additional file 3.** Validation of Affymetrix data by quantitative PCR. Randomly selected genes were analyzed by quantitative PCR. The gene expression ratios (MCTVT to CTVT) of quantitative PCR and Affymetrix are consistent.

**Additional file 4.** Sequences of primers used in quantitative PCR.

## Data Availability

The data supporting the conclusions of this manuscript are included in this article. The datasets used and/or analysed during the current study are available from the corresponding author on reasonable request.

## Abbreviations

CTVT: Canine transmissible venereal tumour
MCTVT: Mouse xenotransplantation of CTVT
XCTVT: Xenotransplanted CTVTs
NOD/SCID: NOD.CB17-Prkdc^scid^/NcrCrl
MHC: Major histocompatibility complex
TILs: Tumour-infiltrating lymphocytes
TME: Tumour microenvironment
PCA: Principal components analysis
DEG: Differentially expressed genes
GO: Gene Ontology
DAVID: Database for Annotation, Visualization, and Integrated Discovery
KEGG: Kyoto Encyclopedia of Genes and Genomes
BP: Biological process
CC: Cellular component
MF: Molecular function
STRING: Search Tool for the Retrieval of Interacting Genes/Proteins
LINE: Long interspersed element
CRC: Colorectal cancer
CMT: Canine mammary tumours
MCODE: Molecular Complex Detection
GSEA: Gene Set Enrichment Analysis
