# Supplementary material for: Canine transmissible venereal tumour established in immunodeficient mice reprograms the gene expression profiles associated with a favourable tumour microenvironment to enable cancer malignancy
Source: BMC Vet Res. 2022 Jan 3;18:4. doi: 10.1186/s12917-021-03093-4 (PMC8722346; doi:10.1186/s12917-021-03093-4)
Supplement: Supplementary file 1 — Additional file 1. The 136 up-regulated genes (≥ 2-fold) in MCTVTs in comparison with CTVTs. [file 12917_2021_3093_MOESM1_ESM.pdf]

Additional file 1. The 136 up-regulated genes (≥ 2-fold) in MCTVTs in comparison with CTVTs.

Probe Set Name	Gene Symbol	Entrez Gene	CP Signal	MP Signal	MP/CP Ratio
Cfa.8843.1.A1_at	APOC1	476437	390.40	2379.00	6.09
Cfa.10996.1.A1_at	LOC480665	480665	172.15	924.10	5.37
Cfa.8843.1.A1_s_at	APOC1	476437	270.60	1385.50	5.12
Cfa.11513.1.S1_at	ERH	480371	217.55	1108.80	5.10
CfaAffx.17453.1.S1_x_at	LOC478576	478576	388.70	1950.05	5.02
CfaAffx.18130.1.S1_at	LOC608732	608732	571.55	2732.20	4.78
CfaAffx.23166.1.S1_at	MMP1	489428	354.75	1429.40	4.03
Cfa.20468.1.S1_at	OTUD5	480911	140.30	522.15	3.72
CfaAffx.2038.1.S1_x_at	LOC475077	475077	1725.00	6410.50	3.72
Cfa.15947.1.A1_at	USP13	478640	127.10	459.65	3.62
Cfa.12478.1.S1_at	GPR177	611491	583.55	2061.80	3.53
Cfa.17131.1.S1_at	FARS2	488204	179.65	633.40	3.53
Cfa.14007.1.A1_x_at	LXN	610062	148.60	503.20	3.39
CfaAffx.20305.1.S1_at	DOCK1	486934	134.25	452.20	3.37
Cfa.16860.1.S1_at	SMARCC2	481107	118.10	389.85	3.30
CfaAffx.15462.1.S1_x_at	LOC474501	474501	4421.80	14574.40	3.30
CfaAffx.15393.1.S1_at	JAM3	489271	149.65	492.10	3.29
Cfa.15489.1.S1_at	RBP4	477775	705.20	2277.00	3.23
CfaAffx.8543.1.S1_at	LOC476453	476453	146.75	470.20	3.20
Cfa.14036.1.A1_at	KMO	480093	280.95	883.70	3.15
CfaAffx.12626.1.S1_at	MICAL2	476858	338.75	1063.95	3.14
CfaAffx.9544.1.S1_s_at	LOC483360	483360	198.45	617.70	3.11
CfaAffx.14398.1.S1_at	CCDC98	478459	119.00	369.25	3.10
CfaAffx.16895.1.S1_at	PDCD4	477818	134.55	414.45	3.08
CfaAffx.21051.1.S1_x_at	LOC478212	476799	185.95	567.30	3.05
Cfa.11612.1.S1_at	UBE4A	479418	3947.80	11993.90	3.04
CfaAffx.345.1.S1_x_at	LOC486372	486372	197.65	599.30	3.03
Cfa.13772.1.A1_x_at	LOC475851	475851	2206.00	6684.25	3.03
CfaAffx.9885.1.S1_at	LOC476183	476183	181.20	545.20	3.01
CfaAffx.7662.1.S1_s_at	SLC6A6	404000	215.90	630.95	2.92
Cfa.14047.1.A1_at	ELAC1	476197	159.70	463.50	2.90
CfaAffx.5753.1.S1_at	FKBP15	481677	288.25	827.55	2.87
CfaAffx.23166.1.S1_s_at	MMP1	489428	493.40	1404.35	2.85
CfaAffx.25660.1.S1_at	GPATCH4	480121	122.05	346.75	2.84
Cfa.3997.1.A1_at	HTR7	477762	117.90	332.25	2.82
CfaAffx.8726.1.S1_s_at	HYI	482531	108.75	306.00	2.81
Cfa.4208.1.S1_at	LOC478181	478181	945.35	2659.60	2.81
Cfa.5692.1.A1_x_at	GNAS	403943	1622.25	4519.85	2.79

Cfa.8070.1.A1_at	LOC475191	475191	288.30	801.75	2.78
CfaAffx.16112.1.S1_at	LOC488054	488054	392.55	1062.95	2.71
Cfa.17009.1.S1_at	LRP1	481124	212.95	553.95	2.60
Cfa.3802.1.S1_s_at	RAB5C	403941	248.80	647.20	2.60
Cfa.15809.1.S1_at	CCL19	448793	183.40	476.55	2.60
CfaAffx.1410.1.S1_s_at	PHACTR2	476230	139.25	360.85	2.59
Cfa.20623.1.S1_s_at	LAMB2	476626	151.80	392.45	2.59
CfaAffx.9682.1.S1_s_at	FMNL2	476151	133.05	343.90	2.58
Cfa.245.1.S1_at	LOC479778	479778	173.65	445.90	2.57
CfaAffx.28854.1.S1_s_at	JUN	609429	153.20	390.50	2.55
CfaAffx.11397.1.S1_at	GAPDHS	476483	178.70	454.85	2.55
Cfa.20474.1.S1_at	ASCC3	475008	219.00	546.05	2.49
CfaAffx.9238.1.S1_at	USP36	483344	141.65	347.10	2.45
Cfa.8772.1.A1_s_at	LOC478722	478722	105.05	257.20	2.45
CfaAffx.30628.1.S1_s_at	SH3GLB2	491306	136.95	332.85	2.43
Cfa.8336.1.A1_at	RSPH3	484057	123.10	296.95	2.41
CfaAffx.25304.1.S1_s_at	LASP1	608624	206.25	497.35	2.41
CfaAffx.20015.1.S1_s_at	TNK2	488025	136.95	329.75	2.41
Cfa.19888.1.A1_at	CEP72	478632	364.50	870.15	2.39
Cfa.1439.1.A1_at	LOC484931	484931	240.90	574.60	2.39
CfaAffx.23127.1.S1_s_at	SLC23A3	488534	128.40	304.05	2.37
Cfa.14652.1.A1_at	DNAJC1	607587	445.35	1053.05	2.36
Cfa.12294.1.A1_at	WDR60	482827	161.00	378.90	2.35
Cfa.21569.1.S1_s_at	HSPG2	403440	138.10	322.35	2.33
CfaAffx.14172.1.S1_at	H3F3A	480110	201.95	470.50	2.33
CfaAffx.23605.1.S1_x_at	LOC479087	479087	150.90	351.50	2.33
Cfa.20329.1.S1_at	PDE4DIP	475817	108.90	253.65	2.33
CfaAffx.13116.1.S1_at	MND1	482653	162.05	376.10	2.32
Cfa.7704.1.A1_s_at	PPRC1	477805	180.65	417.15	2.31
CfaAffx.30111.1.S1_at	ITFG3	490100	123.85	284.15	2.29
Cfa.2263.1.A1_at	DHX29	478060	1498.65	3435.50	2.29
CfaAffx.9661.1.S1_at	LOC612748	612748	204.65	468.70	2.29
Cfa.3834.1.S1_at	C5AR1	442974	224.75	513.10	2.28
Cfa.14036.1.A1_s_at	KMO	480093	189.65	432.15	2.28
CfaAffx.921.1.S1_x_at	LOC477309	477309	550.15	1253.15	2.28
CfaAffx.24956.1.S1_s_at	CASP9	487432	159.65	363.00	2.27
CfaAffx.15444.1.S1_s_at	NKD1	487288	163.65	371.65	2.27
CfaAffx.14981.1.S1_x_at	BAP1	484737	169.75	384.80	2.27
Cfa.21524.1.S1_s_at	BAT2	481713	166.00	376.20	2.27
Cfa.15973.1.A1_at	KHDRBS1	487316	103.70	234.30	2.26
Cfa.19709.2.S1_at	CPNE1	477213	217.05	489.80	2.26
CfaAffx.24184.1.S1_at	PRAF2	480912	241.75	544.80	2.25
CfaAffx.467.1.S1_at	LOC476842	476842	287.50	646.15	2.25

CfaAffx.25435.1.S1_s_at	ZNF688	489908	197.80	443.05	2.24
CfaAffx.15823.1.S1_at	TEX264	476607	298.50	666.55	2.23
CfaAffx.6534.1.S1_at	NARG1	483817	114.95	256.35	2.23
Cfa.20229.1.S1_at	LOC608502	608502	118.35	262.95	2.22
CfaAffx.25378.1.S1_at	ZNF553	489901	125.05	277.65	2.22
Cfa.5955.1.S1_at	CD5L	609953	568.45	1261.60	2.22
CfaAffx.7826.1.S1_s_at	RAB37	483298	143.55	318.15	2.22
CfaAffx.24190.1.S1_at	B3GAT3	483785	188.55	417.45	2.21
Cfa.20160.1.S1_at	FANCD2	484659	177.40	392.05	2.21
Cfa.12122.1.A1_s_at	EMILIN1	475696	187.50	413.80	2.21
Cfa.4210.2.S1_a_at	ATP5J	478393	5880.00	12971.45	2.21
Cfa.40.1.S1_at	IL18	403796	269.25	592.25	2.20
CfaAffx.964.1.S1_x_at	LOC476842	476842	261.10	570.55	2.19
Cfa.17300.1.S1_at	LOC612569	612569	175.65	382.65	2.18
Cfa.10738.1.A1_a_at	RIBC1	480929	107.80	234.70	2.18
Cfa.17841.1.S1_s_at	ACTN4	484526	550.70	1195.95	2.17
Cfa.10174.3.A1_a_at	LOC478370	478370	2090.05	4529.50	2.17
CfaAffx.17515.1.S1_at	GNG4	607513	204.75	443.50	2.17
CfaAffx.29573.1.S1_s_at	PDPK1	479875	290.75	629.60	2.17
Cfa.4292.2.A1_a_at	CIRBP	476755	199.25	429.60	2.16
CfaAffx.30417.1.S1_s_at	LOC489662	489662	178.10	382.05	2.15
Cfa.268.1.A1_at	LOC610276	610276	279.45	597.65	2.14
Cfa.12573.1.A1_at	FKBP15	481677	466.35	997.30	2.14
CfaAffx.3512.1.S1_at	GNMT	474905	195.55	417.95	2.14
CfaAffx.712.1.S1_x_at	LOC485484	485484	624.25	1331.70	2.13
CfaAffx.25462.1.S1_at	CD68	489476	234.05	498.55	2.13
CfaAffx.24794.1.S1_s_at	JARID1C	491894	124.25	264.55	2.13
CfaAffx.681.1.S1_at	LOC610074	479513	321.95	683.40	2.12
Cfa.18316.1.S1_s_at	RNF185	486362	183.85	390.25	2.12
Cfa.16764.1.S1_at	LOC491182	491182	197.25	417.45	2.12
Cfa.10933.1.A1_at	ZCRB1	477640	428.85	906.50	2.11
Cfa.13491.1.A1_s_at	LOC476775	476775	354.85	746.50	2.10
CfaAffx.539.1.S1_x_at	LOC490888	490888	252.20	528.90	2.10
CfaAffx.3764.1.S1_s_at	UBAP2	481583	181.70	380.15	2.09
CfaAffx.22768.1.S1_x_at	LOC478833	478833	112.65	235.40	2.09
CfaAffx.30622.1.S1_s_at	VCAM1	403982	215.60	449.00	2.08
CfaAffx.16370.1.S1_s_at	SH3PXD2A	486874	121.15	252.10	2.08
CfaAffx.14972.1.S1_at	LY86	478712	234.80	488.40	2.08
CfaAffx.8742.1.S1_x_at	HYI	482531	146.50	304.65	2.08
CfaAffx.443.1.S1_x_at	LOC607796	607796	795.15	1652.80	2.08
Cfa.11473.1.A1_at	B3GNT4	486255	125.70	260.75	2.07
Cfa.3362.1.S1_at	DNAJC19	488090	185.50	384.35	2.07
Cfa.13370.1.A1_at	CD68	489476	310.20	637.50	2.06

Cfa.18579.1.S1_at	YBX2	489467	125.15	257.15	2.05
CfaAffx.6706.1.S1_at	FIG4	475023	342.35	702.00	2.05
Cfa.18073.1.S1_s_at	LOC485036	485036	166.00	339.40	2.04
CfaAffx.6342.1.S1_s_at	SCAF1	484376	149.00	303.50	2.04
Cfa.19621.1.S1_s_at	ZNFX1	477260	159.65	324.75	2.03
Cfa.10600.1.A1_at	BAG5	480444	297.70	605.55	2.03
Cfa.15089.1.A1_at	THRA	403601	152.00	308.70	2.03
Cfa.8199.1.S1_at	LOC483462	483462	176.00	357.40	2.03
CfaAffx.29810.1.S1_at	TCF3	485079	180.70	366.75	2.03
Cfa.3913.1.S1_at	PDGFB	442986	282.65	572.90	2.03
CfaAffx.25171.1.S1_at	CAPN8	612029	121.80	246.35	2.02
CfaAffx.14467.1.S1_at	LOC486590	486590	149.40	301.30	2.02
CfaAffx.7814.1.S1_s_at	ZNF342	484453	162.75	328.20	2.02
Cfa.2777.1.A1_at	PHKG1	489784	154.70	311.85	2.02
CfaAffx.30148.1.S1_s_at	QSOX2	607571	150.15	302.35	2.01
Cfa.11701.1.A1_s_at	CCDC63	477479	165.80	333.65	2.01
CfaAffx.8707.1.S1_at	DNAJB6	608937	124.45	249.95	2.01
CfaAffx.4668.1.S1_s_at	EPN1	608964	230.55	461.45	2.00
CfaAffx.16143.1.S1_at	POLS	488057	131.45	263.00	2.00
