# Supplementary material for: Canine transmissible venereal tumour established in immunodeficient mice reprograms the gene expression profiles associated with a favourable tumour microenvironment to enable cancer malignancy
Source: BMC Vet Res. 2022 Jan 3;18:4. doi: 10.1186/s12917-021-03093-4 (PMC8722346; doi:10.1186/s12917-021-03093-4)
Supplement: Supplementary file 2 — Additional file 2. The 37 down-regulated genes (≦ 0.5-fold) in MCTVTs in comparison with CTVTs. [file 12917_2021_3093_MOESM2_ESM.pdf]

Additional file 2. The 37 down-regulated genes (≦ 0.5-fold) in MCTVTs in comparison with CTVTs.

**Probe Set Name**	**Gene Symbol**	**Entrez Gene**	**CP Signal**	**MP Signal**	**MP/CP Ratio**
CfaAffx.20075.1.S1_s_at	MGP	611039	2715.40	752.25	0.28
CfaAffx.23335.1.S1_s_at	SELL	480080	1041.00	292.55	0.28
Cfa.20779.1.S1_at	CXCL12	449622	983.05	284.15	0.29
CfaAffx.1247.1.S1_s_at	VNN1	442973	376.60	114.95	0.31
CfaAffx.15001.1.S1_s_at	SPARCL1	478470	614.35	193.25	0.31
Cfa.4077.1.S1_s_at	CA4	480591	1561.45	495.50	0.32
CfaAffx.13249.1.S1_at	SFRP2	475471	4655.50	1773.65	0.38
CfaAffx.10230.1.S1_at	EEA1	475424	632.40	253.65	0.40
CfaAffx.10118.1.S1_at	POSTN	477298	4576.00	1841.30	0.40
CfaAffx.24909.1.S1_s_at	EMR3	484900	278.70	112.60	0.40
Cfa.1200.1.S1_s_at	SFRP2	475471	4981.75	2016.10	0.40
Cfa.3510.1.S2_at	IL8	403850	1760.25	725.45	0.41
Cfa.11839.1.A1_s_at	PPM2C	477941	453.20	190.05	0.42
CfaAffx.343.1.S1_s_at	HNRPA3	606946	2508.45	1056.50	0.42
Cfa.1490.2.S1_a_at	LOC474612	474612	477.70	201.50	0.42

Cfa.4556.2.S1_at	IGHAC	480452	837.95	359.80	0.43
CfaAffx.20848.1.S1_s_at	HNRPA3	608074	2215.20	976.45	0.44
CfaAffx.12174.1.S1_s_at	EXOC6	477771	1002.20	443.60	0.44
Cfa.16624.1.A1_at	USP38	476071	1046.35	464.05	0.44
CfaAffx.11852.1.S1_at	IPO7	485383	2402.50	1080.40	0.45
CfaAffx.24714.1.S1_at	ACBD3	611888	963.00	444.00	0.46
Cfa.19918.1.S1_s_at	ZNF638	475799	436.15	203.00	0.47
CfaAffx.13200.1.S1_at	NFS1	477214	406.80	189.65	0.47
CfaAffx.13597.1.S1_s_at	VCAN	488922	778.15	363.00	0.47
Cfa.1509.3.A1_s_at	PRPF4B	488199	720.90	339.25	0.47
Cfa.21298.1.S1_s_at	SFRS10	478663	956.25	451.50	0.47
Cfa.16472.2.S1_s_at	C1S	486714	1093.45	517.85	0.47
Cfa.18904.1.S1_s_at	IFI44	490198	262.40	124.30	0.47
Cfa.4077.1.S1_at	CA4	480591	1900.10	905.80	0.48
Cfa.20888.1.S1_s_at	ACTR2	481396	2463.25	1177.75	0.48
Cfa.19109.1.S1_s_at	WDR45L	480820	395.65	190.20	0.48
Cfa.3850.1.S1_s_at	CAV1	403980	2209.95	1063.80	0.48
Cfa.3850.1.S2_at	CAV1	403980	2706.50	1310.55	0.48
Cfa.2663.1.A1_a_at	LOC475941	475941	408.85	199.20	0.49
Cfa.15798.1.S1_s_at	FUT8	448804	431.05	210.10	0.49
CfaAffx.18301.1.S1_s_at	RCC1	487332	853.35	416.80	0.49
Cfa.11921.1.A1_at	AMICA1	610790	358.30	175.30	0.49
CfaAffx.17824.1.S1_s_at	CFI	478515	707.45	347.00	0.49
CfaAffx.28974.1.S1_at	PPAP2B	479557	649.15	318.70	0.49
Cfa.15466.1.S1_s_at	SLU7	479308	664.15	328.00	0.49
CfaAffx.22578.1.S1_at	SLC11A1	478909	325.85	162.35	0.50
