# Supplementary material for: Canine transmissible venereal tumour established in immunodeficient mice reprograms the gene expression profiles associated with a favourable tumour microenvironment to enable cancer malignancy
Source: BMC Vet Res. 2022 Jan 3;18:4. doi: 10.1186/s12917-021-03093-4 (PMC8722346; doi:10.1186/s12917-021-03093-4)
Supplement: Supplementary file 3 — Additional file 3. Validation of Affymetrix data by quantitative PCR. Randomly selected genes were analyzed by quantitative PCR. The gene expression ratios (MCTVT to CTVT) of quantitative PCR and Affymetrix are consistent. [file 12917_2021_3093_MOESM3_ESM.pdf]

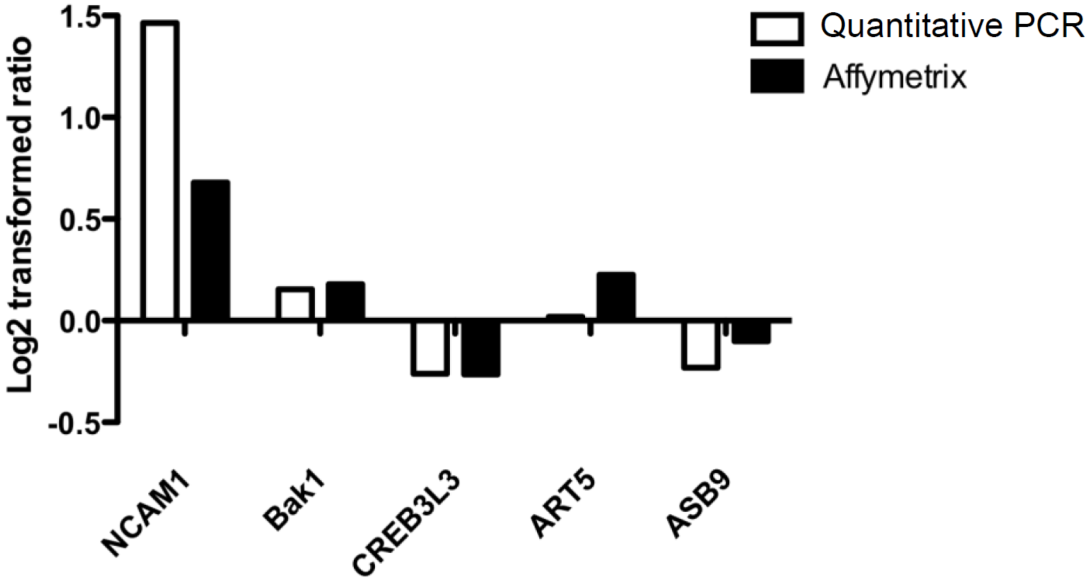

Additional file 3. Validation of Affymetrix data by quantitative PCR. Randomly selected genes were analyzed by quantitative PCR. The gene expression ratios (MCTVT to CTVT) of quantitative PCR and Affymetrix are consistent.
