# Supplementary material for: Canine transmissible venereal tumour established in immunodeficient mice reprograms the gene expression profiles associated with a favourable tumour microenvironment to enable cancer malignancy
Source: BMC Vet Res. 2022 Jan 3;18:4. doi: 10.1186/s12917-021-03093-4 (PMC8722346; doi:10.1186/s12917-021-03093-4)
Supplement: Supplementary file 4 — Additional file 4. Sequences of primers used in quantitative PCR. [file 12917_2021_3093_MOESM4_ESM.pdf]

Additional file 4. Sequences of primers used in quantitative PCR.

Gene symbol	Forward primer	Reverse primer
APOC1	TCTCCCCCTTGAAGAACTGA	ACAGAACCACCACCAAAACC
MMP1	AACGGACTTCAGGCTGCTTA	AACGGACTTCAGGCTGCTTA
KMO	CATGCCATAGTGCCCTTTTT	ATGGCATGGTCATCTGGAAT
CCL19	TTTCTGTAACCCAGCGTCCC	CTGTGGCCCTTGTTCTTTGC
CXCL12	ACCTGCATTTATAGTGTCTGGT	GGCACCGGTGAATATAAGCTG
C5AR1	AGTGAAGGTCTTACTAATCGGGTT	CCGAGGTGCCTAACCAATGT
NCAM1	CGGCATTTACAAGTGTGTGG	CACACAATCACGGCATCTTC
BAK1	ACCCAGAAATGGTCACCTTG	TCATAGCGCTGGTTGATGTC
CREB3L3	GTCCTGCTGTTCTCCTTTGC	GGGTCGTTGTGCAAAGTTCT
ART5	TGTGGGCACTCTTCACTTTG	GAGAGAAGAAGGTGGCGTTG
ASB9	GGGGATATTGTGTCCGATTG	TCTGCTGTGACGAGGTTCAC
β - actin	CCACACCTTCTACAACGAGC	GAACATGATCTGGGTCATCTTCTC
